# Evaluating the Impact of Incentives on Clinical Trial Participation: Protocol for a Mixed Methods, Community-Engaged Study

**DOI:** 10.2196/33608

**Published:** 2021-11-23

**Authors:** Jerome T Galea, Karah Y Greene, Brandon Nguyen, Andrea N Polonijo, Karine Dubé, Jeff Taylor, Christopher Christensen, Zhiwei Zhang, Brandon Brown

**Affiliations:** 1 School of Social Work College of Behavioral and Community Sciences University of South Florida Tampa, FL United States; 2 College of Public Health University of South Florida Tampa, FL United States; 3 Global Health and Social Medicine Harvard Medical School Boston, MA United States; 4 Department of Social Medicine, Population and Public Health University of California, Riverside Riverside, CA United States; 5 Department of Sociology University of California, Merced Merced, CA United States; 6 Gillings School of Global Public Health University of North Carolina at Chapel Hill Chapel Hill, NC United States; 7 HIV+Aging Research Project-Palm Springs Palm Springs, CA United States; 8 Department of Statistics University of California, Riverside Riverside, CA United States

**Keywords:** incentives, ethics, research participation, stakeholder advisory board, HIV

## Abstract

**Background:**

Monetary incentives in research are frequently used to support participant recruitment and retention. However, there are scant empirical data regarding how researchers decide upon the type and amount of incentives offered. Likewise, there is little guidance to assist study investigators and institutional review boards (IRBs) in their decision-making on incentives. Monetary incentives, in addition to other factors such as the risk of harm or other intangible benefits, guide individuals’ decisions to enroll in research studies. These factors emphasize the need for evidence-informed guidance for study investigators and IRBs when determining the type and amount of incentives to provide to research participants.

**Objective:**

The specific aims of our research project are to (1) characterize key stakeholders’ views on and assessments of incentives in biomedical HIV research; (2) reach consensus among stakeholders on the factors that are considered when choosing research incentives, including consensus on the relative importance of such factors; and (3) pilot-test the use of the guidance developed via aims 1 and 2 by presenting stakeholders with vignettes of hypothetical research studies for which they will choose corresponding incentive types.

**Methods:**

Our 2-year study will involve monthly, active engagement with a stakeholder advisory board of people living with HIV, researchers, and IRB members. For aim 1, we will conduct a nationwide survey (N=300) among people living with HIV to understand their views regarding the incentives used in HIV research. For aim 2, we will collect qualitative data by conducting focus groups with people living with HIV (n=60) and key informant interviews with stakeholders involved in HIV research (people living with HIV, IRB members, and biomedical HIV researchers: n=36) to extend and deepen our understanding of how incentives in HIV research are perceived. These participants will also complete a conjoint analysis experiment to gain an understanding of the relative importance of key HIV research study attributes and the impact that these attributes have on study participation. The data from the nationwide survey (aim 1) will be triangulated with the qualitative and conjoint analysis data (aim 2) to create 25 vignettes that describe hypothetical HIV research studies. Finally, individuals from each stakeholder group will select the most appropriate incentive that they feel should be used in each of the 25 vignettes (aim 3).

**Results:**

The stakeholder advisory board began monthly meetings in March 2021. All study aims are expected to be completed by December 2022.

**Conclusions:**

By studying the role of incentives in HIV clinical trial participation, we will establish a decision-making paradigm to guide the choice of incentives for HIV research and, eventually, other types of similar research and facilitate the ethical recruitment of clinical research participants.

**Trial Registration:**

ClinicalTrials.gov NCT04809636; https://clinicaltrials.gov/ct2/show/NCT04809636

**International Registered Report Identifier (IRRID):**

DERR1-10.2196/33608

## Introduction

### Background

Providing incentives, which are defined as “payment[s] (including money, gifts, and services) to research volunteers for participation in studies” [1], is a widely accepted and common practice in HIV clinical research [2-6]. Although some research suggests that altruism is a primary motivation for research participation [3,5,6], incentives are typically necessary for ensuring sufficient participant enrollment in research [7-9], including high-risk trials and other studies that may result in negative health outcomes for participants [10,11]. Although it is known that the incentives provided among similar studies can vary greatly [9,12], little research exists on the factors that are considered important for determining appropriate incentives. This raises concerns about the possibility of unduly influencing participation in research due to the type and quality of the incentives provided [8,13-16].

Many factors influence the type and quality of incentives, including risks, benefits, burdens, historical precedents, study procedures, time commitments, study budgets, institutional review board (IRB) recommendations, advice from other investigators, and local regulations [9,12-14]. In the field of HIV treatment and cure-related research, additional factors regarding incentives are considered, since participants can face greater than minimal risk (eg, the risk of interrupting HIV treatment). In HIV cure research, the outlook for direct individual benefit is low, and participants may face additional social vulnerabilities (eg, belonging to a sexual minority group and having a lower socioeconomic status), which can affect motivations to participate in such research [6,13,16,17]. These factors emphasize the need for ethical incentive decision-making guidelines, especially in biomedical HIV research.

Ideally, incentives encourage participation and participant retention in clinical and behavioral research without causing undue inducement [9,15,18,19]. However, balance for incentive types and amounts can be difficult to establish when significant variability exists across studies. It is important to consider the spectrum of researchers’ attitudes regarding the ethics of incentives as well as beliefs about what IRBs permit. This is reflected by the varying monetary amounts that are approved by IRBs and issued across similar protocols, especially those that are issued at the same institution [9]. Other factors could include study procedures, participants’ setting characteristics (eg, local norms and the cost of living), and variabilities in institutional practices [9].

The documentation of incentive types and amounts in consideration of these factors is lacking [20,21]. The most recent comprehensive study of payment in US-based research (conducted in 2005) described 467 publications of clinical studies, of which fewer than 25% reported payment amounts [9]. Furthermore, a review by Dickert and colleagues [22] showed that less than one-fifth of US institutions knew which of their studies provided payment.

An important topic that will be addressed in our study is the characterization of undue inducement. Even when institutions track payments, significant differences often exist in IRBs’ understanding of undue influence, which is sparsely studied [9,15,21,23]. The Council for International Organizations of Medical Sciences Guidelines state that “[c]ompensation is not meant to compensate for the risk that participants agree to undertake” [24]. However, it is possible that stakeholders (research participants, IRB members, and study investigators) may nonetheless believe that an incentive should compensate for risk and that providing payments is a way of making risks acceptable to participants [3]. We will explore ethical issues related to this topic and consider incentives that may be viewed as too small.

Little is known about the actual effect that incentives have on clinical research participation. In large part, this is due to a lack of comprehensive tracking, a lack of mandates for investigators to record participant payments, and the inconsistent reporting of incentives in published research manuscripts [25-29]. Even in multisite or multinational research, wide variability in incentivizing has been observed. For example, to control over- and underincentivizing, South Africa developed standardized payments for participants [30], while Brazil prohibited the provision of monetary payments for clinical trials [31]. Outside of these rare cases, the absence of a reference for comparison burdens researchers with the need to determine appropriate incentives on a case-by-case basis.

Decisions on acceptable payment should not be made without a clear understanding of currently offered incentives, or else we will continue to develop personal biases that are not critically assessed. Our study aims to lay the groundwork that will guide this emerging area of inquiry toward the establishment of a more systematic study that will develop a framework for determining incentives. Ultimately, we will provide a resource so that decisions on incentive types and amounts are guided by transparent, concrete, and evidence-based decision-making. In the absence of an incentive decision-making tool, it will continue to be impossible to determine the equity of incentives that are offered across similar studies [27].

We are interested to know if stakeholders perceive incentives as a benefit of research participation. Federal law expressly prohibits the consideration of providing compensation to offset risks [1,32]. This is because of concerns that a very risky study may be perceived as having an acceptable risk-benefit ratio simply because it pays a lot of money. It is possible however that participants still consider money to be a benefit of research participation, at least beyond reimbursement. We seek to determine if incentives make the perceived risk-benefit ratio more favorable or acceptable and if they affect the perceived balance of risks and potential benefits [3,33]. There may be ethical issues when incentives sway the decision-making capacities of individuals by making them ignore the risks involved rather than balance the risks and benefits [17,19,33]. We will also ask stakeholders for specific recommendations to improve the description of payments in the informed consent process.

### Specific Aims

The specific aims of our project are to (1) characterize key stakeholders’ views on and assessments of incentives, (2) reach consensus among stakeholders on the factors that are considered important when choosing incentives and on the relative importance of these factors, and (3) pilot-test the use of the guidance developed via aims 1 and 2 by using vignettes of hypothetical research studies. These vignettes will present a variety of HIV research studies that differ in risks, procedures, and incentives.

The projected outcomes from these aims include the determination of different stakeholder groups’ views about incentives, shared decision-making on relevant study factors to consider when deciding on an ethical incentive, and an understanding of how well our chosen factors predict incentive decision-making. We hypothesize that potential study participants make trade-offs based on the characteristics of a research study when deciding on whether to participate. For example, after a study’s risks are weighed against the benefits and incentives offered, a decision to participate will be made.

## Methods

### Overview

**Figure 1:**
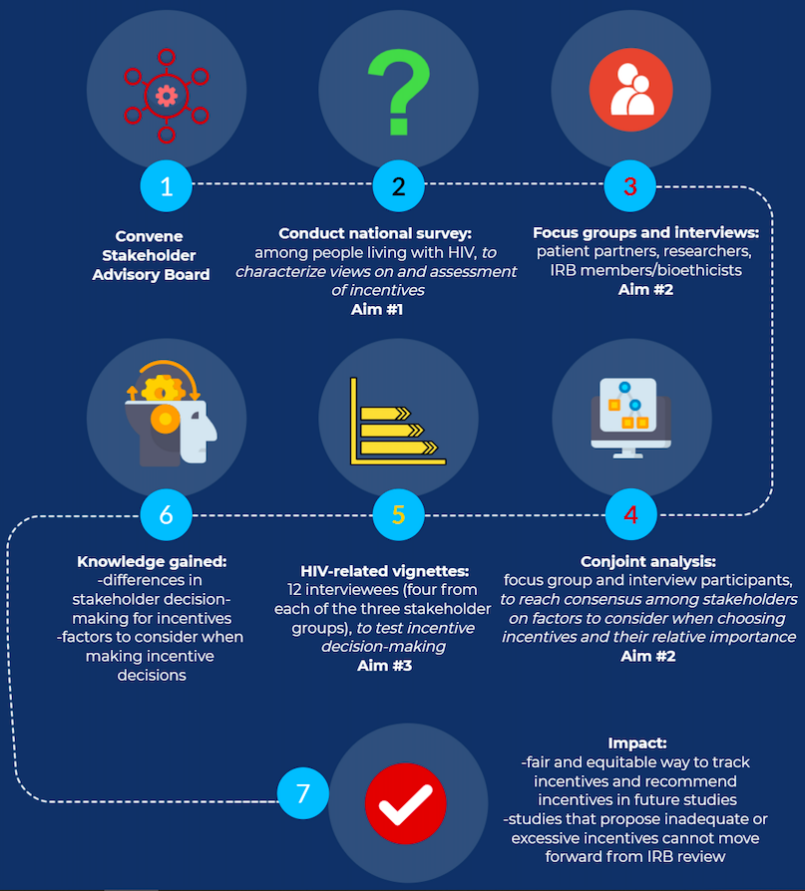
Study aims and process. IRB: institutional review board.

We will use an explanatory, sequential, mixed methods study design to address each study aim. Specific study activities will include the establishment of a stakeholder advisory board (SAB); a national survey of people living with HIV; focus groups and interviews with key stakeholders; a consumer marketing experiment (conjoint analysis [CJA]) to understand the relative importance of different incentive types and amounts in research participation decision-making; and finally, a pilot test of ethical decision-making, which will be conducted by providing case vignettes to stakeholders to determine appropriate incentives (Figure 1). The study will last 24 months (Table S1 in Multimedia Appendix 1).

### Develop an SAB

We will convene a national, 12-member SAB comprised of people living with HIV, IRB members, and HIV researchers to review and provide ongoing feedback and suggestions for the implementation of each study component. The SAB will meet each month via Zoom (Zoom Video Communications Inc) for 90 minutes throughout the study period and receive US $30 for each meeting attended. SAB members will provide their stakeholder perspective to review and amend proposed study materials, help make decisions on study procedures, pilot study activities, and assist with participant recruitment.

### Aim 1: Conduct a National Survey of People Living With HIV

We will conduct a single, internet-based survey with English-speaking US residents who identify as people living with HIV. The 20-minute survey will assess demographic characteristics and how incentives affect the willingness to participate in HIV research. We will include screening questions to ensure that each survey respondent meets our inclusion criteria (Table S2 in Multimedia Appendix 1). Participants who pass the screening questions and complete the survey will be compensated with US $7.

Participants will be recruited through Amazon Mechanical Turk, and the survey will be programmed into Qualtrics (Qualtrics International Inc). Amazon Mechanical Turk is a commonly used platform for recruiting targeted populations and collecting high-quality data, and it is comparable to US web-based survey panels [34-36]. We expect that the purposive national sample will represent people living with HIV who are diverse with respect to their ages, genders, sexual orientations, races and ethnicities, times since diagnosis, and histories of participation in clinical studies. We will analyze survey data by using Stata/SE 17 (StataCorp LLC). We will calculate descriptive statistics for all study variables and use linear, logistic, and multinomial regression models to identify any significant associations between demographic characteristics and key dependent variables.

### Aim 2: Facilitate Focus Groups and Interviews With Key Stakeholders

We will conduct 6 focus groups with people living with HIV (6-10 people per group; up to 60 people in total) to obtain perceptions on the ethics of incentives in research. We especially hope to capture the views of historically underrepresented populations, including older men and women aging with HIV, cisgender and transgender women living with HIV, and adults (aged ≥18 years) of color. We will also focus on recruiting people living with HIV who have comorbid diagnoses of depression, heart disease, and arthritis to ensure that our results are applicable beyond HIV research.

Focus groups will last approximately 1 hour and 40 minutes, will be conducted via videoconferencing, and will be recorded. Participants without sufficient technology for video streaming will be permitted to join discussions via phone. Each participant will receive a US $25 gift card. A key advantage of focus groups is that they help evoke conversations, but they may also foster groupthink [37]. We will mitigate this drawback by having a strong leader who is trained in effective focus group facilitation. By doing so, we will also ensure the receipt of input from all participants, help minimize the amount of irrelevant discussions, and prevent people from speaking over each other. Furthermore, having a strong focus group leader will help transcriptions run smoothly and increase the decipherability of audio data. Our focus group questioning route, as shown in Table S3 in Multimedia Appendix 1, will mirror the key domains in the national survey and the interviews to allow for the triangulation of data. The SAB will review the focus group script prior to IRB submission and implementation.

To obtain perceptions on the ethics of incentives in research, we will conduct 12 key informant interviews (with up to 36 informants in total or until thematic saturation is achieved) with each of the following three stakeholder groups: people living with HIV from across the United States, biomedical HIV researchers, and IRB members and bioethicists. Each interview will last approximately 1 hour, will be conducted via videoconferencing, and will be recorded. Interview participants will receive a US $25 gift card.

Professional informants (eg, biomedical researchers and IRB members and bioethicists) will be individually interviewed one-on-one, as we believe that they will be more likely to share information that they might not openly share with other participants. Key informant interviews have high data yields, are easier to coordinate and transcribe, and provide the flexibility to explore emerging themes while collecting information from knowledgeable individuals [38,39]. We will develop and adapt informed consent forms and interview guides for each category of key informants. The SAB will review the guides prior to IRB submission and implementation.

The main goal of the data analysis will be to generate a list of factors that are deemed the most important to consider when thinking about incentives in HIV research. The qualitative analysis will rely primarily on grounded theory, which seeks to understand the realities grounded in the views of study participants [40]. We will develop a codebook to systematically analyze the data and identify data-driven (or emergent and latent) codes [41]. Code development will be an inductive and iterative process. As relationships among different themes and subthemes become evident, narratives will be combined into general concepts to summarize key informants’ perceptions. We will perform analyses by using MAXDQA software (version 12.1.3; VERBI Software GmbH).

### Aim 2: CJA

CJA is a consumer market-based methodology that was designed to determine the relative “weight” of the characteristics that influence a consumer’s decision to purchase a product or service. CJA follows 2 fundamental assumptions. First, when choosing among very similar products or services, consumers make choices based on the interconnected (ie, conjoined) characteristics that make up products and services and make trade-offs among the characteristics, leading to a product preference. Second, consumers’ product and service preferences are created in a rational way, and consumers preferentially select products and services that increase personal benefits and minimize personal costs. This is referred to as *the theory of random utility maximization* [42]. We consider patient partners to be “consumers” and research studies to be “products” or “services.” When deciding whether to participate in a research study, potential participants make trade-offs among the various study characteristics in a rational way to increase their personal benefits and decrease their personal costs.

CJA is methodologically well suited to help us determine which factors stakeholders consider the most important when choosing incentives and the relative importance of these factors, as this type of analysis can be conducted to efficiently measure the degrees of influence that different factors have on a respondent’s decision-making [43]. Furthermore, CJA has been used to effectively predict preferences for and the acceptability of a wide range of medical services and constructs, including disease treatments and health care systems [44-53]. It has also been used to predict real-world outcomes, such as patients’ actual HIV medication choices [54], and in the assessment of hypothetical biomedical HIV interventions [55,56].

The ability of CJA to accurately reflect and predict consumer preferences is heavily dependent on the type of people participating in the CJA experiment and the selection of study characteristics. The participants in our CJA experiments will be people who have participated or would consider participating in biomedical research, thereby increasing the generalizability of the results to a real-world setting. Each attribute will have different values (referred to as *levels* in CJA) from which participants must choose. For example, the risk characteristic may have 3 levels—no risk, minimal risk, and moderate risk—whereas the incentive received per clinical study interaction may have the values none, US $50, or US $100. The attributes and levels used in our CJA exercises will be determined via extensive consultation with our SAB, people living with HIV, IRB members and bioethicists, and researchers and after a thorough review of relevant research literature.

The selected attributes and levels will be programmed into Sawtooth Software’s choice-based conjoint program (Lighthouse Studio 9, version 9.11.0). Participants in the CJA will be asked to complete an exercise in which they are presented with multiple hypothetical studies or scenarios and must decide whether they would consider participating. Each hypothetical study will be presented as a finite set of attributes that vary in value, as depicted in Table S4 in Multimedia Appendix 1. Participants will demonstrate their preferences for these studies by completing exercises that force trade-offs among similar studies with the same attributes, but the attributes will differ in value across the studies. Scenarios will be presented randomly to prevent order effect bias.

This exercise will allow us to estimate the relative influence that each attribute has on the decisions of each person participating in each hypothetical study. For example, the level of risk associated with participating in a research study may carry a greater “weight” than that of the frequency of required study site visits. Data from the CJA exercises will be used to construct hypothetical scenarios for aim 3.

### Aim 3: Develop HIV-Related Vignettes

By using the data collected via aim 2 and the final number of levels (2-3) per attribute, we will use a factorial design to create 25 hypothetical scenarios (hereafter referred to as *vignettes*). For each vignette, the incentive amounts will not be so large or so small that the answers will be almost unanimous and therefore predictable. The three research incentive amounts that we specify will be reasonable choices. A range of US $0 to US $20,000 is consistent with the studies that we identified in the literature [57-60].

By using the information collected via aims 1 and 2, the SAB will help develop the vignettes. We will contrast different types of HIV studies while being mindful of their parameters, such as study interventions, perceived risks, participant populations, and the inconvenience of study visits. We will also integrate 3 comorbidities (depression, heart disease, and arthritis) into the vignettes to observe differences in decision-making that are beyond the context of HIV. Four key informant interviewees from each of the three stakeholder groups (people living with HIV, IRB members and bioethicists, and researchers: n=12) will pilot-test the 25 vignettes. Participants will select what they believe is the most appropriate incentive for each hypothetical HIV-related vignette (Table S5 in Multimedia Appendix 1). Research participants will receive US $50 as compensation for participating in this portion of the study. A linear mixed model [61] will be used to analyze vignette data, and possible censoring will be handled by using a maximum likelihood approach.

### Demographic Data

Demographic information will be obtained from the national survey respondents, including age, race and ethnicity, the state of residence, and the level of educational attainment. Interview, focus group, and hypothetical study scenario participants will provide demographic data, which will be linked to their confidential responses for the purpose of understanding differences in study data based on key demographic variables. The demographic survey will be completed after consent is provided and immediately before beginning the interviews, focus groups, or hypothetical study scenarios. We will perform standard descriptive, bivariate, and multivariate analyses on survey data.

## Results

We have convened monthly meetings with the SAB since March 2021, and they helped to cocreate all of the study instruments and informed consent documents. SAB members are currently assisting with participant recruitment for focus groups and key informant interviews. We are also collaborating with the SAB to develop the attributes and levels that will be programmed into the CJA software. Data collection and analysis are expected to be completed by December 2022. The dissemination of our findings will be accomplished through conferences, community presentations, the sharing of slide sets, and publications.

## Discussion

Given the lack of guidelines that can assist researchers in ethically incentivizing research participants, it is our hope that the results of our study will establish a paradigm for all future clinical research. Integrating HIV comorbidities into our study will assist in this regard. We will gather pertinent information by interviewing people living with HIV, IRB members, bioethicists, and HIV researchers and by conducting a CJA to determine the relative importance that they place on various study attributes. We will be able to evaluate what factors influence an individual’s decision to participate in a research study by testing decision-making in relation to ethical incentives via HIV-related vignettes. We hope that our findings will provide robust empirical data that will guide future ethical incentive practices in clinical research.

## Appendix

Multimedia Appendix 1 Supplementary tables.

Multimedia Appendix 2 Peer-reviewer report from the Patient-Centred Outcomes Research Institute (PCORI).

## Abbreviations

CJA: conjoint analysis
IRB: institutional review board
SAB: stakeholder advisory board
