# Supplementary material for: Evaluating the Impact of Incentives on Clinical Trial Participation: Protocol for a Mixed Methods, Community-Engaged Study
Source: JMIR Res Protoc. 2021 Nov 23;10(11):e33608. doi: 10.2196/33608 (PMC8663586; doi:10.2196/33608)
Supplement: Multimedia Appendix 1 [file resprot_v10i11e33608_app1.docx]

**Table S1: Project timeline for the 2-year incentives in HIV research study.**

| **Activity** | **Year 1** | | | | **Year 2** | | | |
| --- | --- | --- | --- | --- | --- | --- | --- | --- |
| **Quarters** | 1 | 2 | 3 | 4 | 1 | 2 | 3 | 4 |
| Stakeholder Advisory Board (SAB) meetings | X | X | X | X | X | X | X | X |
| Institutional Review Board review/approval | X |  | X |  |  | X |  |  |
| National survey of people living with HIV |  | X | X |  |  |  |  |  |
| Interviews, focus groups, conjoint analysis |  |  | X | X | X | X |  |  |
| Pilot testing vignettes |  |  |  |  |  |  | X | X |

IRB approval was obtained prior to study initiation, but amendments will be completed to integrate feedback from the SAB on all study materials.

**Table S2: Online questionnaire for people living with HIV.**

| Screener Questions | | |
| --- | --- | --- |
| Question | Answer choices | |
| 1. Spell the word ‘horse’ backwards |  | |
| 2. Describe this picture in 2-3 sentences |  | |
| 3. To ensure you’re paying attention, please select d | 1. the 2. answer 3. is 4. this | |
| 4. Are you a person living with HIV? | 1. yes 2. no | |
| 5. How often do you personally take PrEP? | 1. daily 2. once a week 3. once a month 4. not at all | |
| 6. Which of the following drugs do you usually take for your HIV? | 1. aspirin 2. epinephrine 3. NyQuil 4. none of the above | |
| 7. How were you diagnosed with HIV? (select all that apply) | 1. blood test 2. fluoroscopy 3. electrocardiogram 4. toxicology test. 5. ultrasound 6. saliva test 7. pap smear | |
| 8. Select all the ways that HIV is most likely transmitted | 1. through e-mail 2. sharing toilets 3. condomless vaginal or anal sex 4. Hugging 5. oral sex 6. kissing | |
| Section 1. Demographic Information and Health Status | | |
| 1. What sex were you assigned at birth on your original birth certificate? | 1. Female 2. Male |  |
| 2. How do you describe yourself? | 1. Woman 2. Man 3. Transgender Woman 4. Transgender Man 5. Non-binary or gender queer 6. Something else, specify: __________ 7. I prefer not to answer |  |
| 3. What is your age (in years)?_______________ |  | |
| 4. Which of the following best describes your ethnicity? | 1. Hispanic or Latinx 2. Not Hispanic or Latinx 3. Do not know | |
| 5. Which of the following best describes your race? | 1. American Indian/Alaska Native 2. Asian 3. Native Hawaiian or other Pacific Islander 4. Black or African American 5. White 6. More than one race 7. Other, specify: _______________ 8. Do not know | |
| 6. What is the highest level of education that you completed? | 1. High school 2. Some college 3. Undergraduate degree or equivalent 4. Master’s degree or equivalent 5. Doctorate degree or equivalent terminal degree 6. Other, specify: _______________ | |
| 7. Please tell us the various sources you have for money or support to cover your basic living and other expenses (check all that apply). | 1. Regular, full-time job 2. Regular, part-time job 3. Temporary work 4. Help from family, partners or friends 5. Some form of government support 6. Other, specify: _______________ | |
| 8.  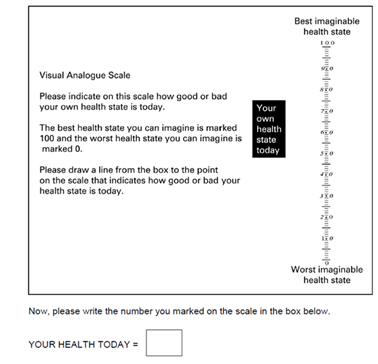 | | |
| Section 2. Study Payment | | |
| 9. Do you consider payment to be a benefit of participating in research? | 1. Yes, please explain___________________ 2. No, please explain____________________ | |
| 10. Do you consider payment to be a benefit of participating in research? | a. Yes, please explain___________________  b. No, please explain____________________ | |
| 11. Would you participate in HIV research without any payment? | a. Yes, please explain___________________  b. No, please explain____________________ | |
| 12. Should people receive payment to participate in HIV research? | a. Yes, please explain___________________  b. No, please explain____________________ | |
| 13. Should there be any standards or policies on participant payment in HIV research? | a. Yes, please explain___________________  b. No, please explain____________________ | |
| 14. If Yes, at what level should these policies be made? | 1. Institution 2. State 3. Federal 4. International 5. Other (specify) | |
| 15. If an HIV study did not pay you, would you expect to receive another benefit from participation? | a. Yes, please explain___________________  b. No, please explain____________________ | |
| 16. Can you imagine a certain risk level from participating in an HIV intervention study in which no amount of payment could convince you to participate? | a. Yes, please specify___________________  b. No🡪 skip to item 18 | |
| 17. If Yes, what % chance of harm, what % chance of death? | 1. % chance of harm_____________ 2. % chance of death_____________ | |
| 18. If one early phase HIV intervention study pays you a total of $20,000, and another early phase HIV intervention study does not pay you, how would you rate the risk of the study? Use a 0-10 scale, with 0 being no risk, and 10 being the highest risk | 1. $20,000 _________ 2. $0 _________ | |
| 19. How should you be compensated for your participation in research? Should it be: | 1. Based on time and wages for your efforts    - 1. Yes      2. No 2. Reimbursement for your expenses, such as mileage, gasoline, or bus fare    - 1. Yes      2. No 3. Based on the study budget    - 1. Yes      2. No    1. Based on burdens and inconvenience to you       1. Yes       2. No 4. Based on something else    - 1. Yes, please specify___________________      2. No | |
| 20. On a scale from 0 to 10, how important are the following if they are offered to you as part of your participation in research? | a. A cash incentive (0-10)  b. Reimbursement for lost wages (0-10)  c. Compensation for your time (0-10)  d. Transportation voucher (0-10)  e. Food (0-10)  f. Gifts (0-10)  g. Post-trial access to the intervention if proven effective (0-10) | |
| 21. Would you like to add anything about payment to participate in research? |  | |
| 22. What is your year of birth? |  | |

**Table S3: Focus group and interview questions.**

| Perceptions of incentives |
| --- |
| 1. Have you ever participated in a research project with an incentive? |
| 2. How do you feel about research incentives? |
| 3. If a research study offers you an incentive to take part, how would that affect your willingness to be a study participant? |
| 4. What are your thoughts about the amount of cash incentives offered to participate in research? |
| 5. How does the offer of an incentive affect your decision to participate in research? |
| Ethical decision-making regarding incentivizing research participants |
| 6. How should decision makers decide on what incentive amounts are appropriate? |
| 7. What factors do you think people (researchers, ethics boards) should consider when making decisions about the type and amount of incentives to offer a study participant? |
| 8. Which form of incentive would you be most interested in receiving in to participate in a research study? |
| Intangible & tangible motivations to participate in clinical research |
| 9. How important do you think it is to participate in research? |
| 10. How does having a doctor invite you to participate in a study affect your willingness to join? |
| 11. How important is the potential for the research to help others on your decision to take part? |

**Table S4: Sample conjoint analysis scenario for decisions on incentive amounts.**

| Characteristic | Value 1 | Value 2 | Value 3 |
| --- | --- | --- | --- |
| Risk Level | None | Minimal | Above minimal |
| Discomfort | None | Some | Lots |
| Location | Walkable | <20 min | <60 min |

**Table S5: Sample vignette to assess appropriate incentive amount.**

| \| Years with HIV  (X) \| Health Status  (Y) \| Study Risk  (Z) \| Study Duration in Years  (AA) \| Income  (BB) \| Co-morbidity  (CC) \| \| --- \| --- \| --- \| --- \| --- \| --- \| \| 1 \| Poor \| High \| 1 \| Low \| Mental health \| \| 5 \| Okay \| Medium \| 2 \| Medium \| Heart disease \| \| 20 \| Excellent \| Low \| 10 \| High \| Arthritis \|   You have been HIV positive for X years. For the past year, your health has been Y. You have the opportunity to participate in a phase II study of HIV cure including an intervention that has proved safe in phase I trials but with unknown efficacy, and with a Z risk of adverse health effects. In order to participate in the trial, you must halt your antiretroviral medications for the duration of the study period AA. Your income is BB and you are living with CC. Would $500 USD be an appropriate incentive to participate? |
| --- | --- | --- | --- | --- | --- | --- | --- | --- | --- | --- | --- | --- | --- | --- | --- | --- | --- | --- | --- | --- | --- | --- | --- | --- |
