# Supplementary material for: Evaluating the Impact of Incentives on Clinical Trial Participation: Protocol for a Mixed Methods, Community-Engaged Study
Source: JMIR Res Protoc. 2021 Nov 23;10(11):e33608. doi: 10.2196/33608 (PMC8663586; doi:10.2196/33608)
Supplement: Multimedia Appendix 2 [file resprot_v10i11e33608_app2.pdf]

PATIENT-CENTERED OUTCOMES RESEARCH INSTITUTE
SUMMARY STATEMENT
(Privileged Communication)

Principal Investigator: Brandon Brown

Organization: Regents of the University of California, Riverside

Project Title: Project DEEPER: DEtermining Ethical Payments in Empirical Research

PCORI Funding Announcement: Improving Methods for Conducting PCOR

Review Cycle: Cycle 1 2018

Request ID: 10879

AVERAGE OVERALL SCORE: 58

QUARTILE: 4

In-Person Review Discussion Notes:

Strengths:

- Reviewers acknowledged the importance of the topic of payments to participants in clinical research.
- The application proposes to use focus groups, interviews, and surveys to gather information from patients and stakeholders, and to apply conjoint analysis and psychological scaling methods to access values and risk-benefit considerations for the various factors related to incentives. This was considered a major strength.
- The proposal demonstrates the principles of reciprocal learning, transparency, and honesty and there is are clear leadership roles for patients and stakeholders.

Weaknesses:

- The approach to and output of creating best practices regarding incentives in a large “town hall” meeting is unclear (Aim 4) and not well justified.
- The generalizability of study results was still a concern since HIV patients’ perspectives on incentives is likely to be very different from other patient groups.
- The outputs and deliverables are not clearly described. It is unclear how the incentive database will be generated, including criteria for selecting studies, and the final product.
- The sample size for the Delphi process is small, which might limit the validity of the estimation of latent variable values from this sample.

The following reviewer critiques were completed prior to the in-person review and were not altered post-discussion.

Criterion 1: Study identifies critical methodological gap(s) in PCOR/CER

Reviewer 1:

Strengths:

- Incentives (payment) for participation in research is a common situation. (moderate)
- There is little clarity in the degree to which any level is considered "appropriate" and not "undue influence." (major)
- This is a methodological gap as incentives are common, but little guidance exists to help researchers and IRBs make decisions. (major)
- Incentives which are too high can create perverse and inappropriate behavior in subjects. (minor)

Weaknesses:

- None noted.

Reviewer 2:

Strengths:

Weaknesses:

Reviewer 3:

Strengths:

- The application identified a critical knowledge gap in the literature on the provision of the appropriate amount of incentives to patients enrolled in medical research (major strength). There is very little reported in the literature on the amount and type of incentive provided to patients, and the limited report reveals dramatic variation by study and institution type.
- The application also convincingly addressed the lack of information on factors that are considered when deciding an appropriate incentive (major strength). Understanding this is important in designing appropriate guidance on research payment for participants.
- The application argues that lack of a clear definition of undue inducement of research participants, as well as, the availability of limited data on guidance on the range, type and amounts of incentives makes it difficult to determine appropriate payments for patients, which is essential to make the right ethical decision on incentive payments for PCOR/CER studies (major strength).

Weaknesses:

- None noted.

Reviewer 4:

Strengths:

Weaknesses:

Reviewer 5:

Strengths:

- Looks to satisfy the criteria for research related to ethical and human subjects protection issues in PCOR/CER. Moderate
- Undue inducement in research is poorly defined based on a review of published guidelines. Moderate
- By the number of grants awarded, this is also a priority topic of the NIH. Minor

Weaknesses:

- The authors claim this work will fill critical gaps concerning patient safety. Learning about the incentive process is not directly related to safety but will allow for a better understanding of the incentive/risk tradeoffs that HIV and HIV subgroup patients are willing to take. Minor

Criterion 2: Potential for the study to improve PCOR/CER methods

Reviewer 1:

Strengths:

- IRBs must make decisions about incentives with no information, and this study will provide several forms of guidance. (major)
- This study will operationalize the phrase "undue incentive" so that clear interpretation can be made about payments. (moderate)
- A database of payments for future reference will be created. (major)
- Incentives affect participant recruitment, and that will be examined here. (moderate)

Weaknesses:

- A table of incentives might be insufficiently complex to be helpful; unless sufficient conditions are included, it might not have relevant factors to apply to all studies. (minor)
- The assumption that incentives are "undue" may not be universal. If an incentive is offered to a person who is comfortably well-off, that may not be compelling. The same incentive to a less-well-off person may create inappropriate levels of behavior. (minor)
- Incentives for certain kinds of research will need to be higher due to research complexity, time in the project, and possible side-effects. (minor)

Reviewer 2:

Strengths:

- A minor strength of the proposal is that, as the proposal states, "If all else fails, researchers will be able to use the transparent database generated from this research for secondary data analysis regarding the cost-effectiveness of incentives in maximizing recruitment and retention, and their impact on the move from efficacy to effectiveness."
- A minor strength of the proposal is that definitions of inadequate, appropriate, and excessive incentives could inform best practices for IRB review of research studies. This is not a moderate or major strength since, as the proposal states, "Consensus definitions of inadequate, appropriate, or excessive incentives may not be reached." Also, unless federal regulations for IRBs are changed to incorporate any study-generated definitions and incentives-related recommendations, usage of any study-generated definitions and recommendations will be limited to a few institutions.

Weaknesses:

- A moderate weakness of the study is that the primary outcome is to come up with definitions of inadequate, appropriate, or excessive incentives. It would be more ambitious to also try to develop one or more formulas for incentives that cover expenses such as travel expenses, time, and study duration.

Reviewer 3:

Strengths:

- The application clearly articulated on the importance of the proposed application for improving the ethical conduct of PCOR/CER studies (major strength). The development of clear incentive guidelines or database (which this study intends to produce) will be used by community members prior to participation, by researchers to plan a budget and by IRB to review protocols.
- Development of payment (incentive) guideline is likely to increase patient participation in PCOR/CER studies (major strength).
- If properly implemented, the current proposed study has the potential to generate data that would be useful in generating payment (incentive) guidelines that will inform best practices on ethical payments for PCOR/CER studies (major strength).

Weaknesses:

- None noted.

Reviewer 4:

Strengths:

- Moderate: The proposed project emphasizes health policy and bioethics.
- Minor: The proposed seeks to create a database that increases transparency on incentives.
- Minor: The project seeks to illuminate the complex issue of incentives as it relates to HIV research, with a focus on excessive incentives and undue inducements.

Weaknesses:

- Major: The validity, trustworthiness, and usefulness of the findings ultimately depend on the quality of data collected. The proposal could be improved by:
  - Describing sampling methods for recruiting survey, focus group, and key informant interview participants (none are articulated).
  - Justifying why qualitative strategies (focus group and interviews) should follow the quantitative survey (typically qualitative findings inform quantitative strategies).
  - Justifying the sample size for the conjoint analysis study (n=15) since standard ranges seem much larger (e.g., 150-1,200), especially considering that the 15 participants may represent up to three different stakeholder groups (this is not specified).
  - Explaining how the 100 studies will be selected for the database (e.g., what criteria will be used to choose 100 studies from the 20,000+ found?).
- Moderate: The narrative includes a critical contradiction regarding the data-driven inquiry. It states that data from Aim 1 will inform Aim 2, but this is contradicted by a subsequent comment that the "EAB will develop all study characteristics that may impact a choice to participate in the study" to inform the development of the conjoint analysis.
- Moderate: The narrative states that "the many co-morbidities associated with HIV, integration of the top 3 co-morbidities (mental health, health disease, arthritis) identified in Dr. Brown's PCORI HIV and aging engagement award into each aim of the study will make the results generalizable to other disease areas." This claim is repeated in many letters of support. The proposal could be improved but including a rationale for this assertion, given that a compelling argument could also be made for these co-morbidities to serve as confounding perspectives. That patient participants will be recruited from a study that focuses on these co-morbidities calls into question whether this approach has true scientific merit or is simply an issue of convenience.
- Minor: The proposal could be improved by defining and carefully using related terms such as "patient partner" and "participant," which are conflated in the narrative.

<u>**Reviewer 5:**</u>

Strengths:

- An incentive database can empower patient engagement and allow an individual to negotiate participation. Moderate
- The database would be used by investigators, community members and the IRB at the study design and approval phase. Moderate
- The database would encourage more public reporting of incentives as guidelines will be more apparent. Minor
- Will better define what is an excessive or undue inducement. Moderate

Weaknesses:

- One could argue that this is not a methodology proposal but a proposal to use information to improve recruitment through best-matched incentives. Minor
- While the team proposed looking at subgroups within the HIV cohort, this is still the only cohort they are using so could impact the generalizability of the results. Minor

Criterion 3: Scientific merit (research design, analysis, and outcomes)

<u>**Reviewer 1:**</u>

Strengths:

- Aim 1: Various forms of surveys, interviews, and focus groups with different types of informants will be used to gather basic information about levels of incentives. (major)
- Compensation to overcome concerns about risk will be considered. (moderate)
- Themes and narratives will be examined to find key rationales for the willingness to accept compensation. (minor)
- Aim 2: Psychometric scaling methods will be used to establish the relative values of study components in the evaluation of incentives. (major)
- Conjoint analysis is a technique which can assess the comparative values and trade-offs of various study components. (moderate)
- Aim 3: Vignettes will be constructed using the information from Aim 2. (minor)
- These will be evaluated by a panel of expert stakeholders using a Delphi process, and assessed using a latent variable approach. The sample size is 15. (minor)
- Aim 4: Best practices for ethical and appropriate incentives will be defined in a large town hall meeting. (minor)

Weaknesses:

- While IRB members and patients will be interviewed, it is not clear that both are at the same level of decision making. Patients agree to participate given certain kinds of incentives. IRB members determine the "undue incentive" of the incentives. It's not clear how IRB members can contribute to this effort, as they may decide that a level of incentive is "undue" while participants might indicate that the same amount is necessary for participation. (major)
- The estimation of latent variable values from such a small sample is very questionable. These techniques have limitations based on the sample size. The confidence bounds on the estimation in the example (p. 10) are very wide and are probably not of practical value. (major)
- The approach to creating best practices in a large "town hall" meeting is not clear. The information from earlier phases should be more directly used. (major)
- The statement that power analysis is not needed is incorrect. Power analysis can be used for estimation, as well as for hypothesis testing. (major)
- The creation of an overall approach to best practices seems quite unworkable. (major)
- A reference is made to a database from the research but the method of constructing this database is not clear. It is not clear what data are in the database, nor how the information is stored. (major)
- The notion of a database may not be particularly sensible since users need algorithms for decision making, not simply collections of data. (moderate)

Reviewer 2:

Strengths:

Weaknesses:

Reviewer 3:

Strengths:

- The idea to determine the study characteristics including incentives when deciding to participate in studies using Conjoint Analysis (a risk-benefit analysis borrowed from marketing) is a major strength.
- In aim 3, the modified RAND appropriateness Delphi method will be used to involve stakeholders from a distance, and this might help to increase the representativeness of the stakeholder participants (moderate strength).

Weaknesses:

- The application lacks a clear ethical, conceptual framework that guides and inform the study design, the collection of key and relevant information and constructs as well as analytic plan (major weakness). Including some basic ethical principles and constructs from bioethics thought leaders would have strengthened the design and the information collection.
- The application does not provide details on the sampling frame. The inclusion of only HIV patient and HIV investigator participants limit the generalizability of the findings and omits diverse perspectives. (moderate weakness). HIV patients represent a very different group and their perspectives on incentives is likely to be very different from other patient groups.
- Although nation-wide quantitative and qualitative methods were suggested, it seems that the study will be recruiting patients through the internet which also limits its generalizability (moderate weakness). Patients who will be recruited through the internet are likely to be different in terms of educational status and other characteristics.
- The questions that investigators plan to ask patients from different parts of the nation are very simplistic and general. They fail to address differences in population characteristics. For example, the cost of living is likely to vary by area and determination of uniform incentives is challenging (moderate weakness). Moreover, the issue of risk is very controversial and more complex than it sounds and cannot really be addressed in these general questions. The inclusion of an interview guide would have helped to evaluate the quality of the data collection instrument. Semi-structured questions for the interview is also not recommended.
- The lack of information on the selection of participants for the Conjoint Analysis is a moderate weakness. Moreover, including all domains and features in a case scenarios study might be very challenging. Participant characteristics also might influence the findings of this type of study and its generalization is questionable (moderate weakness).
- Although the development of scenarios has its own values, the basis on which the Delphi panel makes a determination on whether the incentives are fair or excessive is not provided, and it is unclear how the panel members' judgment will be better than the patients themselves. Most of the panel members are patients and researchers. It would have been better if the guidance is developed by experts in bioethics and Human Subject Protection areas.

<u>Reviewer 4:</u>

Strengths:

Weaknesses:

<u>Reviewer 5:</u>

Strengths:

- Aim 1 will obtain the perspective of patients, researchers and IRB representatives regarding the use of incentives in HIV, depression, heart disease, and arthritis research.
- Aim 1 will use survey, key informant interviews, and focus groups. Moderate
- Appropriate qualitative analysis plan for Aim 1 is provided using grounded theory and the MAXDQA software. Moderate
- Aim 2 will measure the influence that specific incentive amounts have on the decision to participate. Moderate
- The conjoint analysis will be used to assess the relative importance of study characteristics on incentive decisions to participate in a clinical trial. Moderate
- For Aim 3 a factorial design will be used to create 100 vignettes from 8 features and administer them in Delphi Panel to 15 experts. Moderate

- An adaptive Delphi process will be used so all 100 vignettes will not be needed to be used. Moderate
- A latent variable modeling approach will be used to truly reflect the outcomes from the minds of the Delphi panel. Minor
- A program will be designed in Excel for investigators to see how their chosen incentive differs from the best choices from the modeling results. Moderate
- Aim 4 is simply a town hall with investigators to go over the results of the first three aims. Minor

Weaknesses:

- Unique patients with depression, heart disease, and arthritis will be not be used rather co-morbid subgroups within the HIV cohort will participate in Aim 1. These patients may not be representative of all patients with depression, heart disease, and arthritis. Moderate
- Examples of survey questions would help in understanding how this will be quantitative data analysis. Perhaps Yes/No or multiple choice is being used. Minor
- It isn't until Aim 3 that a literature review is implemented for incentives. This seems like a beginning step. Minor
- Not sure what is gained from the Aim 4 town hall that you will not get from the quarterly EAB meetings. Minor
- Outputs and deliverables are never clearly described. It is unclear what the incentive database will look like. A simple transcription of the town hall does not provide a guideline document. Moderate
- Conjoint analysis appendix is too theoretical and does not relate to the project. Moderate

Criterion 4: Investigator(s) and environment

Reviewer 1:

Strengths:

- The PI is an experienced researcher in the HIV area and has experience with ethical issues. (major)
- The Co-I is experienced in mixed-methods research which will be used to identify themes. (moderate)
- A biostatistician is associated with the project. (moderate)
- Other HIV experts are associated with the project. (moderate)

Weaknesses:

- It is not clear who the conjoint analysis methodologist is. (moderate)

Reviewer 2:

Strengths:

Weaknesses:

Reviewer 3:

Strengths:

- Dr. Brandon Brown (PI) is trained in epidemiology and international health and works on the area of HIV. In

conjunction with the PI, the investigative team is capable of implementing the proposed study (major strength).
- The level of effort for each team member is appropriate, and the research environment is outstanding (major strength).

Weaknesses:

- The research team would have benefited by having additional bioethics expertise.

Reviewer 4:

Strengths:

Weaknesses:

Reviewer 5:

Strengths:

- Dr. Brown, the study PI is an epidemiologist with a career in HIV and other STD research with over 80 publications. He will provide project oversight, be present for interviews and focus groups, help construct the vignettes, and oversee the EAB assignments. He is at 25% effort. Major
- Dr. Dube, co-I, is a faculty social scientist at Chapel Hill and is the expert in mixed methods and HIV research and shew ill conduct the focus groups and interviews and conduct survey. She will be at 20% effort. Major
- Dr. Galea, co-I is also an HIV researcher and social scientist with a community focus, he will write the interview and focus group questions and will be in charge of the conjoint analysis at 10% effort. Moderate
- Dr. Zhang, co-I, is the team biostatistician and will be 5% in year 1 with increasing effort as the study progresses. Major
- Drs. Eyal and Coulter will serve as consultants with Coulter being a Delphi panel expert and Eyal as the bioethicist. Moderate
- Jeff Taylor will serve as the patient partner with an annual stipend of $500. Major
- The team comes from UC Riverside, UNC, Harvard, U of South Florida, and UCLA, so the proposal shows a collaborative effort of researchers in the field and the team covers all the needed expertise. Major
- While all the team's institutions provide facility access, much of the work is done offsite and virtually. Moderate

Weaknesses:

- None noted.

Criterion 5: Patient-Centeredness

Reviewer 1:

Strengths:

- This application discusses the best way to compensate patients, which is patient-centered. (major)
- The grant discusses the ethical aspects of compensating patients which is strongly patient-centered. (major)

Weaknesses:

- None noted.

Reviewer 2:

Strengths:

Weaknesses:

- A moderate weakness of this proposal is that the results may not improve methods that address outcomes of interest to patients and caregivers. This reviewer feels that the results will be of little interest to patients and caregivers unless the study's results lead to an outcome of changes in IRB regulations; that outcome is uncertain. Patients have differing opinions about incentives. A release of definitions about different categories of incentives will not have a direct impact on patients and their care in clinical studies unless the definitions are incorporated into new regulations.

Reviewer 3:

Strengths:

- The study is clearly patient-centered as it attempts to develop payment guidelines which will help minimize undue inducement and exploitation of patients (major strength).
- The study also has the potential to improve PCOR/CER methods by increasing participation, thus increasing the validity of the findings (major strength) which is of interest to patients and caregivers.

Weaknesses:

- The application and investigators failed to articulate how the study is addressing outcomes that are important to patients and caregivers. Investigators are confusing patient-centeredness with patient engagement (minor weakness).

Reviewer 4:

Strengths:

- Moderate: This work is novel, representing the first comparative study of financial incentives for patient participation in clinical research.

Weaknesses:

- Moderate: The stated hypothesis is that "investigators (particularly junior investigators) who propose incentives and then view our database will change their views and accept suggestions for incentives proposed. While investigators may continue to choose similar incentives following creation and pilot testing of the incentive framework, the proposed study will create a product that can be used as a reference point to evaluate incentives in future studies." Given the application's policy framing, this seems a relatively weak hypothesis. The study could be improved by providing in some guidance/summary document for IRBs.

- Lead Patient Stakeholder Jeff Taylor's letter of support states, "The procedures you have outlined in this study will achieve several important outcomes: including understanding how patients view and assess incentives, developing a method to support ethical incentive decision making, and the creation of a policy regarding incentive justification and transparency." Two of the three are not reflected in the protocol ("a method to support ethical decision-making" and "the creation of a policy"). In fact, the narrative provides a candid look at challenges, noting that, "If all else fails, researchers will be able to use the transparent database generated from this research for secondary data analysis regarding cost-effectiveness of incentives in maximizing recruitment and retention, and their impact on the move from efficacy to effectiveness."

Reviewer 5:

Strengths:

- The author proposes that this is a patient-centered and driven project because patient partners will provide direction and input on progress. Minor
- Patients will be part of interviews and focus groups. Minor
- Patients will benefit in so far as incentives will tie to study intensity (level of risk, length of study, number of follow-up periods, amount of data collected at each visit, etc.) based on the guidelines established with the project. Moderate

Weaknesses:

- This proposal does not directly impact patient care nor does it affect the decision making ability of the clinicians so is only tangentially patient-centered. Moderate
- The team seems to confuse the PCORI definition of patient-centeredness to mean patient involvement in the study rather than whether the work proposed will improve patient care and decision making. Moderate

Criterion 6: Patient and Stakeholder engagement

Reviewer 1:

Strengths:

- Three stakeholder groups will be used to inform the team about the issues. (major)
- Stakeholders will be involved at different levels. (major)
- IRB members, who make these judgments of ethical levels of compensation, are particularly important and are members of the stakeholders. (major)

Weaknesses:

- None noted.

Reviewer 2:

Strengths:

- A major strength of the proposal is that the study will strive to include a group of patient partners that is diverse

with regard to age, gender, sexual orientation, race/ethnicity, time since diagnosis, and history of participation in clinical trials. They are also going to recruit at least 200 patient partners so it is likely that they will meet their goal of assembling a diverse group.

- A major strength of the proposal is that the study will engage with a variety of stakeholders - patient partners, biomedical researchers, and IRB members - and involve them throughout all of the stages of the research project.
- A moderate strength of the proposal is that the study will use a variety of methods for engagement - surveys, interviews, and focus groups. This is not a major strength due to the fact that the number of patient partners and biomedical researchers who will be included in in-depth interviews, twelve each, could be higher.

Weaknesses:

- None noted.

Reviewer 3:

Strengths:

- The involvement of patients, researchers, and IRB members as part of the patient and stakeholder group is a moderate strength.
- The presented plan indicates that the stakeholders will participate at all levels of the implementation of the study (moderate strength).

Weaknesses:

- The omission of experts in bioethics in the stakeholder engagement is a major weakness.

Reviewer 4:

Strengths:

- Major: Table 5 (page 12) lays out roles for all stakeholder groups (patients, biomedical researchers, and IRB members) and provides a rationale for the one area patients are not included.
- Major: Principles of reciprocal relationships, co-learning, partnership, trust, transparency, and honesty are embedded throughout the proposal. Examples include a leadership role for a key patient representative and full access to data for all stakeholders.
- Moderate: All External Advisory Board (EAB) members receive identical compensation for their contributions ($150/meeting).
- Moderate: The dissemination plan is structured to maximize data and analytic transparency among participating stakeholders and the public. Funds are allocated to support patient stakeholder travel to a key discussion/dissemination meeting.

Weaknesses:

- Moderate: Patient stakeholders and participants will be recruited from research underway in the Palm Springs/Coachella Valley region of California, which has a focus on gay men ages 50+. This narrow demographic focus may translate to a narrower patient perspective that would be ideal for this type of research.

- Minor: The narrative could be improved by better distinguishing between stakeholders and participants. Particularly among engaged patients, these roles tend to be conflated and thus confusing.

Reviewer 5:

Strengths:

- There will be three stakeholder groups: patients, researchers, and IRB members). Major
- An external advisory board (EAB) will be created and have quarterly meetings with the investigators. Moderate
- Participants in each Aim of the study are stakeholder patients, researchers, and IRB representatives, so stakeholder participation extends well beyond the EAB. Major

Weaknesses:

- None noted.

Does the application have acceptable risks and/or adequate protections for human subjects?

Reviewer 1:

Yes

Reviewer 2:

Reviewer 3:

Yes
Appropriate IRB approvals will be sought.

Reviewer 4:

Reviewer 5:

Yes

Overall Comments:

Reviewer 1:

The objective assessment of research incentives such as compensation is an understudied component of research. Participation in research projects by subjects is contingent upon incentives, and some of these include monetary compensation. Setting these assessments correctly is a complex process, as incentives are needed (of various sorts), and little guidance is available for the levels of these incentives. Incentives must not have "undue influence" on participants to encourage participation in an overly risky study. This is a research gap. This application suggests a rational evaluation of the level of incentives as a characteristic of studies. The project will begin by surveying participants and running focus

groups involving participants, IRB members, and stakeholders who run studies. These will assess levels of incentives. Conjoint analysis, a scaling technique, will be used to provide a valuation to different components of the incentive situation. Vignettes of compensation scenarios will be constructed, and the Dephi process will be used to This is an interesting proposal, which suggests a scientific approach to the issue of compensation. The sample size for the Delphi scaling step is small, and the confidence bounds for the simulated data are very large, so large that there are questions as to their value. N=15 is a small sample size. The final aim involves generating best practices in a large town hall meeting which seems confused and incoherent. Such venues are ill-suited for complex policy formation, and there is no guarantee that information obtained in earlier steps will be incorporated. There is a reference to a database, but the clarity in generating this database is absent. The guidance for IRBs in using the information is missing. The project has some value, but the final step is not well designed. There are unresolved questions, such as the differential attraction of incentives based on participant income level, and on the cost of living in the research area.

Reviewer 2:

Comments on methodology: How will they locate the informed consent forms for review? Will they use a convenience sample? This is not entirely clear.
Study aims
Aim 1: Characterize how key stakeholders view and assess incentives using formative mixed methods
Will use surveys, interviews, and focus groups.
Aim 2. Determine the primary study characteristics considered when deciding to participate in biomedical research and the relative importance that each characteristic, including incentives, has on the decision to participate.
Aim 3. Develop a framework to support ethical incentive decision making
Aim 3 has several parts, including: i) creating vignettes, ii) the Delphi process, and iii) creating a model for payment.
They will create a database of incentives data for 100 studies.

"Once we have our final model, and a database of results of the vignettes from the Delphi panel including the factors and incentive amounts, we will create a macro (framework) in Microsoft Excel, which is an automated form of recording a series of tasks. Site investigators for 20 in-development biomedical HIV treatment and HIV cure studies will pilot test the framework for incentive decision making (Figure 4). Investigators will propose incentives without the framework; then enter their study parameters into the framework. Once they have the framework results, which will provide the range of incentives in similar studies and suggested cutoff points, investigators can choose to either revise their incentives or leave them as originally established. Initial and final incentives will be reported back to the research team, as will any concerns or difficulties investigators have had in using the macro for incentive decision making. We will examine the variance between initial incentives and framework results to identify numerical changes. Investigators will be asked if and how the framework modified their decision making, which will help refine the framework for future data collection. A separate survey will be provided to investigators to catalog incentives from the 100 studies in preparation for the Delphi panel as inadequate, appropriate, or excessive, with room to categorize incentives in 'gray areas'. This categorization of incentives will help inform the larger discussion with all stakeholders on what incentives are appropriate in various contexts of research risks and patient factors.

Aim 4. Create best practices regarding incentives
"Consensus definitions of inadequate, appropriate, or excessive incentives may not be reached, and balancing what works in research with what is ethical can be challenging."

Criteria 2
A moderate weakness of the study is that the primary outcome is to come up with definitions of inadequate, appropriate, or excessive incentives. It would be more ambitious to also try to develop one or more formulas for incentives that cover expenses such as travel expenses, time, and study duration.

Criteria 5
A moderate weakness of this proposal is that the results may not improve methods that address outcomes of interest to patients and caregivers. This reviewer feels that the results will be of little interest to patients and caregivers unless the study's results lead to an outcome of changes in IRB regulations; that outcome is uncertain. Patients have differing opinions about incentives. A release of definitions about different categories of incentives will not have a direct impact on patients and their care in clinical studies unless the definitions are incorporated into new regulations.

Criteria 6
A major strength of the proposal is that the study will strive to include a group of patient partners that is diverse with regard to age, gender, sexual orientation, race/ethnicity, time since diagnosis, and history of participation in clinical trials. They are also going to recruit at least 200 patient partners, so it is likely that they will meet their goal of assembling a diverse group.

A major strength of the proposal is that the study will engage with a variety of stakeholders - patient partners, biomedical researchers, and IRB members - and involve them throughout all of the stages of the research project.

A moderate strength of the proposal is that the study will use a variety of methods for engagement - surveys, interviews, and focus groups. This is not a major strength due to the fact that the number of patient partners and biomedical researchers who will be included in in-depth interviews, twelve each, could be higher.

Reviewer 3:

The application attempts to identify factors affecting decision-making in setting payments (incentives) for patients participating in medical research. Investigators made a convincing argument that there is a critical knowledge gap on how payment decisions for research participants (regarding amount and type) are made and what factors are involved in the decision-making process. Moreover, the investigators argued on the importance of clearly defining undue inducement of research participants and the need for guidelines to decide the type and amount of incentives for various studies. The proposed study clearly has the potential to improve the ethical conduct of studies and may inform the development of payment (incentive) guidelines that are likely to increase patients' participation--thus increasing the validity of PCOR/CER studies. Other strengths of the application include: (i) the utilization of Conjoint analysis and the Delphi panel; (ii) experienced researchers in the field of HIV; (iii) patient-centered study; and (iv) a reasonable stakeholder engagement plan. On the other hand, the application lacks a clear ethical, conceptual framework to guide the study design, and analyses and details are missing on the sampling frame. The study instrument is simplistic and fails to accommodate differences in participants' characteristics. Other weaknesses include (i) recruitment of participants through the internet; (ii) lack of expertise in bioethics in the Delphi panel; (iii) non-generalizability of findings; (iv) the absence of a co-investigator with bioethics expertise; and (v) failure to articulate how the proposed study is patient-centered. Because the weaknesses outweigh the strengths of the application, the study's potential to exert sustained and powerful influence on PCOR/CER studies is in question.

Reviewer 4:

The proposed study aims to create a model to inform ethical incentive decision making based on input from diverse stakeholders to (1) illuminate perspectives related to research incentives (2) develop a data-driven framework for decision-making on this topic and (3) create policy. The research question is novel and highly relevant through a lens of bioethics and policy. The project's greatest strength is its commitment to stakeholder engagement, with patients, researchers, and IRB experts assigned meaningful roles spanning the entire research process. However, the proposal lacks overall systems thinking and key details across the proposal that raise serious concerns for this stakeholder reviewer, particularly as they relate to Criteria 2 and 5. Lack of detail and/or justification in several areas (e.g., recruitment and sample size) ultimately call into question the project's ability to deliver data of sufficient quality to inform the type of policy framework envisioned.

Reviewer 5:

The goal of this study is to prepare a database of preferred incentives for different levels of risk, pain, and other study factors based on patient interviews, vignette reviews by researchers using the Delphi panel, literature review and a Town Hall type discussion of findings. The study involves stakeholders throughout the process both as subjects and as part of the External Advisory Board. The team is excellent, bringing together the best folks from several institutions to fill the needed roles. The design is also we constructed in terms of the structure of the focus groups, interview surveys and the analytic approach to each aim. While this research does not directly affect patient care and clinician decision making it will hopefully improve future trials by minimizing dropouts dues to inappropriate incentives. They have added substantially to the proposal the case for the need for further research in this area and that NIH is also supporting such research.

The team was asked to address the generalizability of the study given the use of only HIV patients, they provide a few references and decide to look at HIV subgroups with depression, heart disease, and arthritis. It is not clear that this is sufficient and they do not address the sample size issues when subgroups become the primary analysis group. HIV is the research focus of almost all of the team members, so it is logical that this group of patients is used. Other concerns include the late addition of the literature review in the study, the poor reference for conjoint analysis in Appendix A, and lack of example questions. It is also unclear what is really gained from the Town Hall Aim 4 above and beyond the prior three aims, and there is no clearly defined output from this Aim or the study in general.
